# An Herbal Galactagogue Mixture Increases Milk Production and Aquaporin Protein Expression in the Mammary Glands of Lactating Rats

**DOI:** 10.1155/2015/760585

**Published:** 2015-05-05

**Authors:** Haibin Liu, Ying Hua, Hui Luo, Zhaojun Shen, Xuejiao Tao, Xueqiong Zhu

**Affiliations:** ^1^Department of General Surgery, The Second Affiliated Hospital of Wenzhou Medical University, Wenzhou 325027, China; ^2^Department of Obstetrics and Gynecology, The Second Affiliated Hospital of Wenzhou Medical University, Wenzhou 325027, China

## Abstract

*Background*. Herbal galactagogues have been increasingly used to treat postpartum hypogalactia. The mechanism of action of herbal galactagogues remains unclear. The purpose of this study was to investigate the effect of an herbal galactagogue mixture on milk production and aquaporin (AQP) expression in lactating rats. *Methods*. Thirty female Sprague Dawley rats were randomized into virgin, lactating + H_2_O, and lactating + galactagogue groups (*n* = 10 per group). Lactating rats were administered the decoction of an herbal galactagogue mixture by oral gavage or the same amount of distilled water. *Results*. The herbal decoction significantly increased milk production in lactating rats (*P* < 0.05). Both immunohistochemical staining and western blot showed that protein levels of AQP-3 and AQP-5 were significantly increased during lactation compared with virgin stage and the herbal decoction further elevated their expression (*P* < 0.05). AQP-1 was predominantly expressed in the capillaries whereas AQP-3 and AQP-5 were mainly detected in the epithelial cells and ducts of the mammary glands. *Conclusion*. The expression of AQPs in the mammary glands of rats was developmentally regulated. Herbal galactagogues might have increased milk secretion by regulating the expression and function of AQPs in the mammary glands.

## 1. Introduction

Breast milk is considered the best source of nutrient for babies, particularly for newborns. However, many mothers suffer from postpartum hypogalactia because of emotional stress, anxiety, and maternal illness [1]. Winikoff and Baer reported that approximately 20–40% of mothers willing to breastfeed experienced postpartum hypogalactia [2]. In recent years, the rate of postpartum hypogalactia is expected to rise continuously possibly due to substantial increases in maternal age and the rate of cesarean section, particularly in the metropolitan area of China. To relieve postpartum hypogalactia, improvements of breastfeeding technique, for instance, increasing baby's proper sucking reflex and initiating early breastfeeding, are often recommended [3]. Pharmacological therapies such as metoclopramide, oxytocin, and domperidone are sometimes used as well, but safety concerns associated with these therapies have limited their use [4–6].

Herbal galactagogues are increasingly used as alternative therapies to enhance breast milk production. Sim et al. conducted a survey in Western Australia to investigate the use of herbal medicine in women who were breastfeeding and found that 24% of survey respondents used herbal galactagogues such as fenugreek and blessed thistle during breastfeeding [7]. Some studies show that herbal galactagogues can increase breast milk production [1, 8, 9]. The attitude of breastfeeding women toward herbal galactagogues was quite positive [10]. In a randomized controlled study on 358 Chinese breastfeeding women with postpartum hypogalactia, Guo et al. found that the decoction of an herbal galactagogue mixture significantly increased breast milk secretion compared with the control group [11]. Studies using animal models showed that extracts of herbal galactagogues, such as Silitidil which is a standardized extract of milk thistle and Silymarin BIO-C which is an extract from* Silybum marianum* fruits, increased serum prolactin levels significantly in female rats [12, 13]. However, the molecular mechanism underlying the action of herbal galactagogues remains unknown.

Aquaporins (AQPs), a family of membrane proteins facilitating water movement across cell membrane, are expressed in the mammary glands, suggesting that they might play roles in milk secretion [14–16]. It has been shown that a distilled extract from* Astragalus membranaceus*, which is commonly used as an herbal galactagogue, can regulate AQP-4 expression [17]. Thus, herbal galactagogues might affect milk production by regulating the function and expression of AQPs. Here, to test this hypothesis, we investigated the effects of an herbal galactagogue mixture on AQP expression in mammary tissue and milk production in lactating rats.

## 2. Materials and Methods

### 2.1. Reagents

Rabbit anti-rat antibodies for AQP-1, AQP-3, and AQP-5 were bought from Sigma-Aldrich (St. Louis, Missouri, USA). The recipe of the herbal galactagogue mixture has been commonly used to treat postpartum hypogalactia in China and was described in the previous study [11]. In brief, the herbal mixture contained 10 g* Vaccaria hispanica* seed, 15 g* Euphorbia heterophylla*, 9 g pangolin scales, 15 g* Astragalus membranaceus*, 6 g* Tetrapanax papyriferus*, 12 g* Platycodon grandiflorum*, and 15 g* Cnicus benedictus*. Information regarding the chemical constituents of* Euphorbia heterophylla*,* Astragalus membranaceus*, and* Cnicus benedictus* can be found on the website of Sigma-Aldrich (http://www.sigmaaldrich.com/china-mainland/zh/life-science/nutrition-research/learning-center/plant-profiler.html). The chemical constituents of other plants in the galactagogue mixture are not available. The mixture was boiled in 200 mL distilled water and then decocted for 1 hour to reach a final volume of 87 mL. The concentration of the herbal mixture in the final decoction was 1 g/mL. The herbal decoction was stored in refrigerator for future use.

### 2.2. Animals

Female Sprague Dawley rats (6–8 weeks old, body weight of 200–250 g) were purchased from Slac Laboratory Animal Ltd. (Shanghai, China). All procedures of animal care and experiments have been approved by the Institutional Ethics Committee for Animal Care and Usage of Wenzhou Medical University. The rats were randomized into the following 3 groups (*n* = 10 per group): virgin group, lactating + H_2_O group in which lactating rats were administered distilled water starting from postpartum day 1, and lactating + galactagogue group in which lactating rats were administered the herbal decoction starting from postpartum day 1. The herbal decoction was administered by oral gavage at 2 mL/kg body weight 3 times (7–10 hours interval) per day for 8 days. The dose level was determined based on the dose level used in patients and adjusted proportionally according to the body weight ratio of rats versus human beings. For lactating + H_2_O group, the same amount of distilled water was administered. The rats in the virgin group were sacrificed at the same time to collect the mammary tissues. The rats in the other 2 groups were mated at the same time and became pregnant at similar time. Milk production and collection of mammary tissues were conducted at similar time for the 2 groups.

### 2.3. Quantification of Milk Production

Quantification of milk production was performed according to the previous description [18]. The amount of milk production was determined by comparing the body weight of rat pups before and after feeding. At postpartum day 3, the pups were removed from their mother and starved for 4 hours. The body weight of the pups after starving was measured using an EB-3200D Shimadzu electronic balance (Kyoto, Japan). The pups were then returned to the mother allowing for feeding for 1 hour and then removed from the mother again. The body weight of the pups was measured again immediately. The difference in body weight of pups before and after feeding was used to represent the amount of lactation. The starve-feeding cycle was repeated 3 times per day and the amount of milk production was accumulated for each day. Milk production was measured daily from postpartum day 3 to day 7.

### 2.4. Immunohistochemistry

At postpartum day 8, lactating rats were anesthetized by an intraperitoneal injection of 0.1% sodium pentobarbital (40 mL/kg) and then sacrificed by cervical dislocation. Virgin rats were sacrificed at 8 weeks of age. Mammary tissues from 5 rats in each group were dissected, fixed immediately in 10% formalin solution for 24 hours, and embedded in paraffin. The mammary tissues were then sliced into 4 *μ*m sections. The mammary tissue sections were deparaffinized, rehydrated, immersed in 3% H_2_O_2_ for 10 min, washed with phosphate-buffered saline, and blocked with normal goat serum (10% in PBS) at 4°C overnight. The tissue sections were then incubated with primary antibodies rabbit anti-rat AQP-1 (1 : 200), AQP-3 (1 : 200), or AQP-5 (1 : 200). After washing thoroughly, the tissue sections were incubated with biotinylated secondary antibody and then streptavidin peroxidase was added to develop color. Tissue sections exposed to secondary antibody only were used as negative controls. Images of immunohistochemical staining were collected using an Olympus optical microscope.

### 2.5. Western Blot

Rat mammary tissues from 5 rats in each group were dissected and homogenized. Proteins were extracted in ice-cold RIPA lysis buffer containing protease inhibitors. Protein concentration in the lysate was measured by bicinchoninic acid assay (Pierce Biotechnology, Rockford, USA). 50 *μ*g protein lysates were loaded on 12% of sodium dodecyl sulfate polyacrylamide gel for electrophoresis and then transferred to polyvinylidene fluoride membrane. The membrane was blocked with 5% dry milk in Tris-buffered saline containing 0.1% Tween 20 (20 mM Tris/HCl, 137 mM NaCl, and 0.1% Tween 20, pH 7.5) for 2 hours at room temperature. After blocking, the membrane was incubated with rabbit anti-rat antibodies for AQP-1 (1 : 200), AQP-3 (1 : 200), AQP-5 (1 : 200), and mouse anti-rat *α*-tubulin (1 : 2000, Santa Cruz, CA, USA) at 4°C overnight. *α*-tubulin was used as the loading control. After washing, the membrane was incubated with the secondary antibodies (Santa Cruz, CA, USA) for 1 hour at room temperature. Western-Bright ECL Plus Enhanced chemiluminescence reagent (GE Healthcare, NJ, USA) was used to visualize the target proteins. The experiment was repeated 3 times. The density of protein signals on the membrane was quantified by the densitometry analysis software Alpha Ease FC.

### 2.6. Statistical Analysis

Continuous data were presented as mean ± standard deviation. For normally distributed data, difference among multiple groups was analyzed by one-way ANOVA. LSD method was used to compare 2 groups. All statistical analyses were conducted using the statistical analysis software SPSS 17.0 (Chicago, IL, USA). *P* value was 2-sided and *P* < 0.05 was considered statistically significant.

## 3. Results

### 3.1. The Galactagogue Mixture Increased Rat Lactation

**Figure 1:**
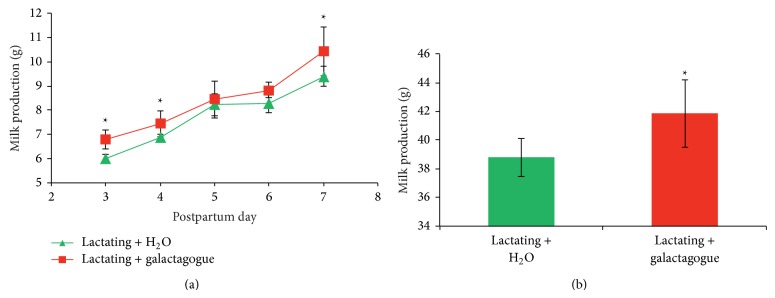
The decoction of the herbal galactagogues increased milk production in lactating rats. (a) Milk production from postpartum day 3 to day 7. (b) Total accumulated milk production from postpartum day 3 to day 7. The amount of daily milk production from postpartum day 3 to day 7 was added. ∗ represents significant difference between lactating + H_2_O and lactating + galactagogue; *P* < 0.05.

Lactating rats were administered the galactagogue mixture from postpartum day 1 to day 8. Milk production in lactating + galactagogue group was significantly increased at postpartum days 3, 4, and 7 compared with that in lactating + H_2_O group (all *P* < 0.05, Figure 1(a)). The accumulated milk production from postpartum day 3 to day 7 was also significantly higher in lactating + galactagogue group than in lactating + H_2_O group (*P* < 0.05, Figure 1(b)).

### 3.2. The Galactagogue Mixture Increased the Expression of AQP-3 and AQP-5 in Mammary Tissue

**Figure 2:**
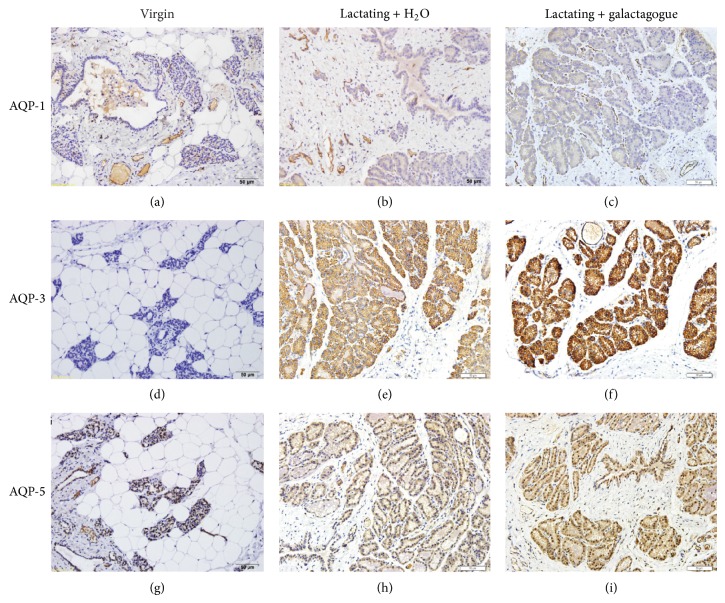
Immunohistochemical staining of AQPs in rat mammary tissue. Lactating rats with or without herbal galactagogue treatment at postpartum day 8 and 8-week-old virgin rats were sacrificed. The tissue sections were then stained with rabbit anti-rat antibodies for AQP-1, AQP-3, and AQP-5. Images of immunohistochemical staining (200x) were collected using an Olympus optical microscope system (Japan).

We next examined the expression of AQPs in mammary tissue. Immunohistochemical staining showed that 8-week-old virgin rats expressed abundant AQP-1. The staining signals of AQP-1 were predominantly around capillaries but weak in epithelial cells and mammary ducts (Figure 2(a)). The levels of AQP-1 expression in lactating rats (Figures 2(b) and 2(c)) at postpartum day 8 were similar to those in virgin rats (Figure 2(a)). In contrast to the abundant expression of AQP-1, the expression of AQP-3 was not detected in virgin rats (Figure 2(d)) whereas it dramatically increased in lactating rats (Figures 2(e) and 2(f)). The staining signals of AQP-3 were in mammary epithelial cells and ducts. Compared with lactating + H_2_O group (Figure 2(e)), AQP-3 expression in lactating + galactagogue group was increased markedly (Figure 2(f)). These data suggest that AQP-3 expression in rats is upregulated during lactation and can be further stimulated by the galactagogue mixture. Both AQP-1 and AQP-3 were expressed in the cytoplasm. Similar to AQP-1, the expression of AQP-5 was abundant in virgin rats (Figure 2(g)). The staining signals of AQP-5 were mainly in the nuclei but not in the cytoplasm in virgin rats but appeared in both cytoplasm and nuclei of mammary epithelial cells and ducts in lactating rats (Figures 2(g)–2(i)). The levels of AQP-5 were increased considerably in lactating rats (Figures 2(h) and 2(i)) compared with virgin rats (Figure 2(g)). In addition, AQP-5 expression was higher in lactating + galactagogue group (Figure 2(i)) than in lactating + H_2_O group (Figure 2(h)).

**Figure 3:**
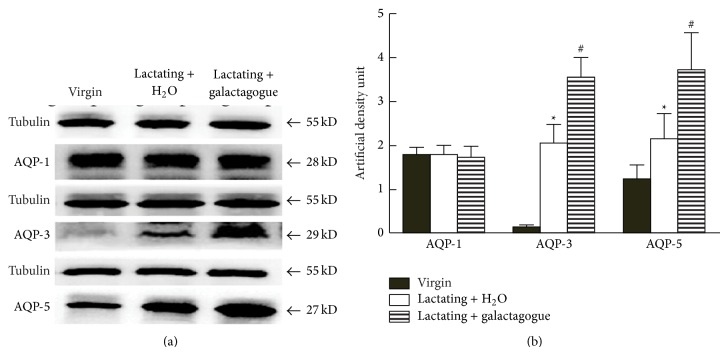
Western blot of AQPs in rat mammary tissue. (a) Western blot of AQPs in rat mammary tissue. (b) Quantification of the western blot. The protein signals were quantified by measuring the density of the signals on the western blot. ∗ represents significant difference between lactating + H_2_O and virgin rats. # represents significant difference between lactating + galactagogue and lactating + H_2_O. *P* < 0.05.

To quantitatively evaluate the effects of the galactagogue mixture on AQP expression, we performed western blot. Consistent with the immunohistochemical staining, AQP-1 protein level was similar in the 3 groups, whereas AQP-3 and AQP-5 were significantly upregulated in lactating rats compared with virgin rats (*P* < 0.05, Figure 3). In addition, the levels of both AQP-3 and AQP-5 were further elevated significantly by the galactagogue mixture (*P* < 0.05, Figure 3).

## 4. Discussion

In this study, immunohistochemical staining revealed that AQP-3 and AQP-5 were expressed in mammary epithelial cells and ducts, whereas the staining signals of AQP-1 were mainly in capillaries of mammary tissue of lactating rats. These staining results are similar to the findings from studies on AQP expression in rat, mice, and bovine mammary gland [15, 16]. Additionally, consistent with our findings showing the absence of AQP-3 in the mammary tissue of virgin rats, Matsuzaki et al. did not detect AQP-3 in virgin rats or mice either [15]. AQP-1 and AQP-3 have also been detected in human mammary glands [19–22]. We also found that, in contrast to AQP-1 and AQP-3, which were predominantly expressed in cytoplasm, the subcellular localization of AQP-5 was exclusively present in nuclei in virgin rats whereas during lactation it was detected in both cytoplasm and nuclei. Nazemi et al. also found that the immunohistochemical staining signals of AQP-5 were in mammary epithelial cells and duct system in rats but the signals were mainly in cytoplasm [23]. Both our study and the report by Nazemi et al. show that the staining intensity of AQP-5 was lower than that of AQP-3 in lactating mammary glands.

The exact function of AQPs in milk secretion is still unclear. Results in this study and previous findings by others suggested that AQPs in mammary gland are developmentally regulated. Thus, the function of AQPs might evolve at different stages of mammary gland development including virgin, pregnancy, lactation, and involution. In this study, immunohistochemical staining and western blot demonstrated that protein levels of AQP-3 and AQP-5 were significantly increased in lactating rats compared with virgin rats. Nazemi et al. investigated the expression of AQP-1, AQP-3, and AQP-5 during pregnancy and lactation in rats and found that, compared with pregnancy stage, the mRNA levels of AQP-1 and AQP-3 were significantly increased at lactation stage [23]. Similar to the results in this study, they demonstrated significantly higher protein levels of AQP-3 at lactation than at pregnancy [23]. In our study, the upregulation of AQP-3 at lactation was dramatic considering the fact that it was absent in virgin rats. Nazemi et al. also found the largest increase in both mRNA and protein levels of AQP-3 at lactation compared with pregnancy. These findings indicate that AQP-3 might play a critical role in milk secretion. During lactation, substantial amount of triglyceride is synthesized and secreted from mammary tissue [24]. In addition to water, AQP-3 is also an essential protein for glycerol transport [25]. AQP-3 might participate in glycerol uptake and transportation during milk secretion. A study on AQP expression during the reproduction cycle of Tsetse fly demonstrated that AQP expression was increased during milk secretion and that knock-down of AQP resulted in an increase in milk osmolarity [26]. These results suggest that AQPs might be possibly involved in the maintenance of milk osmolarity during storage in the mammary duct system.

We also found that the decoction of the herbal galactagogue mixture significantly increased milk production in lactating rats and stimulated the protein expression of AQP-3 and AQP-5 in the mammary glands of lactating rats. The effects of herbal galactagogues on AQP expression have also been demonstrated in a study on ischemia-induced cerebral edema. Li et al. found that astragaloside IV, which is an extract from an herbal galactagogue* Astragalus membranaceus*, significantly inhibited cerebral edema-associated overexpression of AQP-4 [17]. In this study, we did not find any lesions in mammary tissue that could be associated with toxicity effect; however, long-term safety of the herbal galactagogue mixture remains to be determined in future studies. Since the herbal galactagogue mixture has been commonly used in China for several decades and no safety concerns have been reported, the herbal mixture most likely seems to be safe.

## 5. Conclusions

In summary, we found that, in the mammary glands of lactating rats, AQP-1 was mainly localized around capillaries, whereas AQP-3 and AQP-5 appeared in epithelial cells and ducts. The protein levels of AQP-3 and AQP-5 were significantly increased in lactating rats compared with virgin rats. To the best of our knowledge, we first discovered that the decoction of the herbal galactagogue mixture significantly increased the protein levels of AQP-3 and AQP-5 in the mammary glands of lactating rats, suggesting that herbal galactagogues might increase breast milk production by regulating AQPs.
